# Supplementary material for: Diagnostic accuracy of GeneXpert MTB/RIF assay for detection of tubercular pleural effusion
Source: PLoS One. 2021 Jun 14;16(6):e0251618. doi: 10.1371/journal.pone.0251618 (PMC8202948; doi:10.1371/journal.pone.0251618)

PLOS ONE

Diagnostic accuracy of GeneXpert MTB/RIF assay for detection of tubercular pleural effusion

--Manuscript Draft--

**Manuscript Number:**	PONE-D-19-33113
**Article Type:**	Research Article
**Full Title:**	Diagnostic accuracy of GeneXpert MTB/RIF assay for detection of tubercular pleural effusion
**Short Title:**	" GeneXpert MTB/RIF assay and tubercular pleural effusion."
**Corresponding Author:**	jyoti jain, M.D.<br>mahatma gandhi institute of medical sciences<br>Wardha, Maharashtra INDIA
**Keywords:**	diagnostic accuracy; GeneXpert MTB/RIF assay; Tuberculosis; Tubercular pleural effusion; sensitivity; specificity; Positive predictive values; Negative predictive values; Positive likelihood ratios; Negative likelihood ratios.
**Abstract:**	Background: India has been engaged in Tuberculosis (TB) control activities for over 50 years and yet TB continues to remain India's important public health problem. The present study was conducted to compare the performance of GeneXpert MTB/RIF (GXpert) assay with composite reference standard in diagnosing cases of tubercular pleural effusion (TPE) and to evaluate the reliability of rifampicin resistance.Settings and Design: A cross-sectional study was performed in a Department of Medicine of a rural teaching tertiary care hospital in central India.Materials and Methods: In all consecutive patients with pleural effusion on chest radiograph presenting to Department of Medicine, GXpert assay and composite reference standard was performed to evaluate the diagnostic accuracy of GXpert assay for detecting TPE in comparison to composite reference standard.Statistical Analysis Used: Standard formulae were used to calculate the sensitivity, specificity, positive predictive values (PPV), negative predictive values (NPV), positive likelihood ratios (LR+) and negative likelihood ratios (LR-). Mc-Nemar's test was applied to compare variables. All comparisons were two-tailed. We considered the difference to be statistically significant if the P value was less than 0.05.Results: The sensitivity of the GXpert assay in diagnosing TPE was 16.6%, the specificity was 100% and diagnostic accuracy was 52.5% which was statistically significant (p value < 0.05).It had a PPV of 100% (95%CI: 88.3% - 100%) and a NPV of 52.4% (95%CI: 39.3% - 55.7%). The LR+ and LR-were 23.5 (95%CI: 1.4- 38.6) and 0.83 (95%CI: 0.76- 0.91) respectively.Conclusion: GXpert assay has a very high specificity in diagnosing TPE but has a low sensitivity. In comparison to composite reference standard Thus its clinical utility is limited when used as a standalone test. A physician's clinical acumen in combination with routine pleural fluid analysis should be the key factor in the diagnosis of TPE in clinically and radiologically suspected patients, especially in high TB burden countries.
**Order of Authors:**	Jyoti* Jain, M.D.
	Pooja$ Jadhao
	SHASHANK^ SHARADCHANDRA BANAIT
	Preetam# Salunkhe
**Opposed Reviewers:**	
**Additional Information:**	
**Question**	**Response**
**Financial Disclosure**<br><br>Enter a financial disclosure statement that describes the sources of funding for the work included in this submission. Review	The author(s) received no specific funding for this work.

Powered by Editorial Manager® and ProduXion Manager® from Aries Systems Corporation

the submission guidelines for detailed requirements. View published research articles from *PLOS ONE* for specific examples.<br><br>This statement is required for submission and **will appear in the published article** if the submission is accepted. Please make sure it is accurate.<br><br>**Unfunded studies**<br>Enter: *The author(s) received no specific funding for this work.*<br><br>**Funded studies**<br>Enter a statement with the following details:<br>• Initials of the authors who received each award<br>• Grant numbers awarded to each author<br>• The full name of each funder<br>• URL of each funder website<br>• Did the sponsors or funders play any role in the study design, data collection and analysis, decision to publish, or preparation of the manuscript?<br>• **NO** - Include this sentence at the end of your statement: *The funders had no role in study design, data collection and analysis, decision to publish, or preparation of the manuscript.*<br>• **YES** - Specify the role(s) played.<br><br>* typeset	
**Competing Interests**<br><br>Use the instructions below to enter a competing interest statement for this submission. On behalf of all authors, disclose any competing interests that could be perceived to bias this work—acknowledging all financial support and any other relevant financial or non-financial competing interests.<br><br>This statement **will appear in the published article** if the submission is accepted. Please make sure it is accurate. View published research articles from *PLOS ONE* for specific examples.	The authors have declared that no competing interests exist.

Powered by Editorial Manager® and ProduXion Manager® from Aries Systems Corporation

**NO authors have competing interests**<br><br>Enter: *The authors have declared that no competing interests exist.*<br><br>**Authors with competing interests**<br><br>Enter competing interest details beginning with this statement:<br><br>*I have read the journal's policy and the authors of this manuscript have the following competing interests: [insert competing interests here]*<br><br>* typeset	
**Ethics Statement**<br><br>Enter an ethics statement for this submission. This statement is required if the study involved:<br><br>• Human participants<br>• Human specimens or tissue<br>• Vertebrate animals or cephalopods<br>• Vertebrate embryos or tissues<br>• Field research<br><br>Write "N/A" if the submission does not require an ethics statement.<br><br>General guidance is provided below. Consult the submission guidelines for detailed instructions. **Make sure that all information entered here is included in the Methods section of the manuscript.**	The study was approved by the ethics committee of Mahatma Gandhi Institute of Medical Sciences (IRB00003623). We obtained a written informed consent from all study participants before enrolling them in the study.

**Format for specific study types**<br><br>**Human Subject Research (involving human participants and/or tissue)**<br>• Give the name of the institutional review board or ethics committee that approved the study<br>• Include the approval number and/or a statement indicating approval of this research<br>• Indicate the form of consent obtained (written/oral) or the reason that consent was not obtained (e.g. the data were analyzed anonymously)<br><br>**Animal Research (involving vertebrate animals, embryos or tissues)**<br>• Provide the name of the Institutional Animal Care and Use Committee (IACUC) or other relevant ethics board that reviewed the study protocol, and indicate whether they approved this research or granted a formal waiver of ethical approval<br>• Include an approval number if one was obtained<br>• If the study involved *non-human primates*, add *additional details* about animal welfare and steps taken to ameliorate suffering<br>• If anesthesia, euthanasia, or any kind of animal sacrifice is part of the study, include briefly which substances and/or methods were applied<br><br>**Field Research**<br><br>Include the following details if this study involves the collection of plant, animal, or other materials from a natural setting:<br>• Field permit number<br>• Name of the institution or relevant body that granted permission	
**Data Availability**<br><br>Authors are required to make all data underlying the findings described fully available, without restriction, and from the time of publication. PLOS allows rare exceptions to address legal and ethical concerns. See the PLOS Data Policy and FAQ for detailed information.	Yes - all data are fully available without restriction

A Data Availability Statement describing where the data can be found is required at submission. Your answers to this question constitute the Data Availability Statement and **will be published in the article**, if accepted.<br><br>**Important:** Stating 'data available on request from the author' is not sufficient. If your data are only available upon request, select 'No' for the first question and explain your exceptional situation in the text box.<br><br>Do the authors confirm that all data underlying the findings described in their manuscript are fully available without restriction?	
**Describe where the data may be found in full sentences. If you are copying our sample text, replace any instances of XXX with the appropriate details.**<br><br>• If the data are **held or will be held in a public repository**, include URLs, accession numbers or DOIs. If this information will only be available after acceptance, indicate this by ticking the box below. For example: *All XXX files are available from the XXX database (accession number(s) XXX, XXX.*).<br>• If the data are all contained **within the manuscript and/or Supporting Information files**, enter the following: *All relevant data are within the manuscript and its Supporting Information files.*<br>• If neither of these applies but you are able to provide **details of access elsewhere**, with or without limitations, please do so. For example:<br><br>*Data cannot be shared publicly because of [XXX]. Data are available from the XXX Institutional Data Access / Ethics Committee (contact via XXX) for researchers who meet the criteria for access to confidential data.*<br><br>*The data underlying the results presented in the study are available from (include the name of the third party*	If the data are held or will be held in a public repository, include URLs, accession numbers or DOIs. If this information will only be available after acceptance, indicate this by ticking the box below.

and contact information or URL).<br>• This text is appropriate if the data are owned by a third party and authors do not have permission to share the data.<br><br>* typeset	
Additional data availability information:	Tick here if the URLs/accession numbers/DOIs will be available only after acceptance of the manuscript for publication so that we can ensure their inclusion before publication.

Powered by Editorial Manager® and ProduXion Manager® from Aries Systems Corporation

To,

The Editor

PLOS One

Dear Sir,

We intend to publish an article entitled "**Diagnostic accuracy of GeneXpert MTB/RIF assay for detection of tubercular pleural effusion**." in your journal as a **research article**. In the light of our findings we have reached to the conclusion that GXpert assay has a very high specificity in diagnosing tubercular pleural effusion. In high TB burden countries like India although its clinical utility is limited when used as a standalone test, however combining it with clinical diagnosis will improve the diagnosis of tubercular pleural. In our study we found the sensitivity of the GXpert assay in diagnosing tubercular pleural effusion to be 16.6% and the specificity to be 100%. These values were similar to earlier reports which found a low sensitivity and high specificity of GXpert assay in detecting tubercular pleural effusion.

No any prior interactions with PLOS regarding the submitted manuscript.

I am suggesting **Sarman Singh,** orcid.org/0000-0002-0749-9647 from All India Institute of Medical Science – Bhopal, INDIA as Academic Editor for this manuscript and no any opposed reviewers

Thanking you,

Dr. Jyoti Jain **Corresponding contributor:**

Department of Medicine, Mahatma Gandhi institute of medical sciences

Sevagram, Wardha, Maharashtra - 442102

India

E-mail address – jyotijain@mgims.ac.in

Manuscript
Click here to access/download;Manuscript;research paper pooja to send .docx

Diagnostic accuracy of GeneXpert MTB/RIF assay for detection of tubercular pleural effusion

Jyoti jain[1¶*], Pooja Jadhav[1¶], Shashank Banait[2&], Preetam Salunke[1&]

[1]Department of Medicine, Mahatma Gandhi Institute of Medical sciences, Sewagram, Wardha, Maharashtra, India.

[2]Department of Ophthalmology,Jawaharlal Nehru Medical College, Sawangi (Meghe), Wardha, Maharashtra, India.

[*]Corresponding author

E-mail: jyotijain@mgims.ac.in

¶These authors contributed equally to this work.

&These authors also contributed equally to this work.

Abstract

Background: India has been engaged in Tuberculosis (TB) control activities for over 50 years and yet TB continues to remain India's important public health problem. The present study was conducted to compare the performance of GeneXpert MTB/RIF (GXpert) assay with composite reference standard in diagnosing cases of tubercular pleural effusion (TPE) and to evaluate the reliability of rifampicin resistance.

Settings and Design: A cross-sectional study was performed in a Department of Medicine of a rural teaching tertiary care hospital in central India.

Materials and Methods: In all consecutive patients with pleural effusion on chest radiograph presenting to Department of Medicine, GXpert assay and composite reference standard was performed to evaluate the diagnostic accuracy of GXpert assay for detecting TPE in comparison to composite reference standard.

Statistical Analysis Used: Standard formulae were used to calculate the sensitivity, specificity, positive predictive values (PPV), negative predictive values (NPV), positive likelihood ratios (LR+) and negative likelihood ratios (LR-). Mc-Nemar's test was applied to compare variables. All comparisons were two-tailed. We considered the difference to be statistically significant if the P value was less than 0.05.

*Results**:*** The sensitivity of the GXpert assay in diagnosing TPE was 16.6%, the specificity was 100% and diagnostic accuracy was 52.5% which was statistically significant (p value < 0.05).It had a PPV of 100% (95%CI: 88.3% - 100%) and a NPV of 52.4% (95%CI: 39.3% - 55.7%). The LR+ and LR- were 23.5 (95%CI: 1.4- 38.6) and 0.83 (95%CI: 0.76- 0.91) respectively.

*Conclusion***:** GXpert assay has a very high specificity in diagnosing TPE but has a low sensitivity. In comparison to composite reference standard Thus its clinical utility is limited when used as a standalone test. A physician's clinical acumen in combination with routine pleural fluid analysis should be the key factor in the diagnosis of TPE in clinically and radiologically suspected patients, especially in high TB burden countries.

Introduction

Tuberculosis is one of the oldest diseases known to affect humans (1). India has been engaged in TB control activities for over 50 years and yet TB continues to remain India's important public health problem as it kills about 480,000 Indians every year (2, 3).

Tubercular pleural effusion is the second commonest form of extra pulmonary tuberculosis after TB lymphadenitis (4). Major challenges in the diagnosis of extra pulmonary TB are its atypical clinical presentation, difficulties in obtaining specimens and the paucibacillary nature of the disease (5, 6). Demonstrating the organism in pleural fluid by conventional microbiological techniques (staining and/or culture) have long been the cornerstone in diagnosing TPE(5). These techniques are largely inadequate as the sensitivity of smear examination can be as low as 1%, the results of culture are too slow to help in clinical decision-making (7). In high TB burden settings, diagnosis of TPE is concluded by exudative pleural fluid with positive adenosine deaminase (ADA) levels along with lymphocytic predominance (8). However, false-negative and false-positive results are a challenge.

In early 2011, World health organization (WHO) endorsed a novel, rapid, automated, molecular diagnostic test, the GXpert assay, which can detect both *Mycobacterium tuberculosis* (MTB) complex and rifampicin resistance within 2 hours (9-11). Li et al emphasized that compared with acid fast bacilli (AFB) smear and solid culture, GXpert assay has high sensitivities and short detection time, so could be used as an alternative for the rapid diagnosis of extra pulmonary TB in clinical practice.(12) However, Sehgal et al. (13) in a systematic review have strongly revoked the recommendations by WHO stating that neither the comparison of GXpert assay with culture as reference standard is justified (due to the low yield of culture in TPE) nor the comparison of GXpert assay with a composite reference standard is justified as GXpert assay has low sensitivity (22.7%).

The excellent diagnostic performance of GXpert assay has been demonstrated in sputum sample but not many studies have been undertaken for the evaluation of extra pulmonary samples. The

present study was conducted to compare the performance of GXpert assay with composite reference standard in diagnosing cases of TPE and to evaluate the reliability of rifampicin resistance.

Materials and Methods

Ethics

The study was approved by the ethics committee of Mahatma Gandhi Institute of Medical Sciences (IRB00003623). We obtained a written informed consent from all study participants before enrolling them in the study.

Setting

The study was conducted in the department of Medicine, Mahatma Gandhi Institute of Medical Sciences, Sevagram which is a 1000-bedded teaching tertiary care hospital located in a town in central India. There are approximately 4, 50,000 outpatient visits and about 12,000 patients are admitted each year in department of Medicine in this Hospital. All consecutive patients with pleural effusion on chest radiograph were screened among patients visiting Medicine OPD or patients admitted under Medicine Department.

Study Design

Between October 1, 2016 to March 31, 2018 a cross-sectional observational, hospital based study was conducted among all consecutive patients with pleural effusion on chest radiograph visiting Medicine OPD or patients admitted under Medicine Department. Patients with minimal pleural effusion which could not be tapped, history of bleeding diathesis or other contraindications to pleural fluid tapping and patients not willing to participate in the study were excluded.

Sample size

Sample size calculated for the study was 158. This was based on sensitivity of 54.8% of GXpert assay for pleural TB and specificity of 100% along with 80% power of the study and alpha error of 5% [11].

All study subjects with radiological evidence of pleural fluid were subjected to clinical, haematological and biochemical work up after detailed history and clinical examination. The management of the study subjects was carried out as per standard guidelines. We studied the complete blood count and other biochemical parameters like serum proteins and serum lactate dehydrogenase (LDH) required for Light criteria.

Processing of pleural fluid samples:

At the first visit of the study subjects, was tapped in all patients and the pleural fluid samples were subjected to index and reference tests simultaneously. Each sample was centrifuged and the pellet was aliquoted into parts as follows: 1 ml for GXpert assay, pleural fluid ADA, culture on Löwenstein–Jensen (LJ) medium, AFB on microscopy, pleural fluid LDH and pleural fluid protein for future reference. The initiation of anti-tubercular treatment (ATT) was not delayed pending results of GXpert assay. All patients were followed up at monthly intervals or on a required basis until completion of ATT.

GXpert assay on pleural fluid samples (Index test):

The GXpert assay was carried out using GeneXpert IV system (Cepheid Inc., Sunnyvale, CA, USA) as per the manufacturer's instructions. Briefly, to the 1 ml of sample, 2 ml of sample reagent was added. The mixture was vortexed for 30 seconds, then left to stand for 15 minutes

and finally added to the cartridge. The total turn-around time per sample was 1-2 hours and it was done free of cost. Standard myco-bacteriological procedures were followed the results to be estimated after which they were displayed on the computer screen and the results were distinguished among the following: no TB; TB detected, rifampicin resistance detected; TB detected, no rifampicin resistance detected; TB detected, rifampicin resistance indeterminate; and an invalid result.

Composite Reference standard on pleural fluid samples:

Composite reference standard was used to evaluate the diagnostic accuracy of a GXpert assay in the absence of a perfect reference test. Composite reference standard included following tests: Exudative pleural fluid (by Lights criteria) with positive pleural fluid ADA level (Sensitivity 90-100% and a Specificity 89-100%) and/or positive for AFB on microscopy and/or positive culture on LJ medium and/or response to treatment. According to Light's criteria, the fluid is considered an exudate if: ratio of pleural fluid to serum protein greater than 0.5; ratio of pleural fluid to serum LDH greater than 0.6 or pleural fluid LDH greater than two thirds of the upper limits of normal serum value. The standardized protocol for pleural fluid ADA was used, as has been previously published and pleural fluid ADA levels greater than 42U were considered positive.

Ziehl Neelsen (ZN) staining for smear microscopy of pleural fluid following the WHO recommended protocol was done. Patients with positive ZN smear of pleural fluid or growth of MTB on Mycobacterial Growth Indicator Tube (MGIT) liquid medium were also considered as cases of TPE.

All the study subjects considered as cases of TPE by exudative pleural fluid (by Lights criteria) with positive pleural fluid ADA level and/or Positive for AFB on microscopy were initiated on anti-

tubercular treatment with four drugs- Isoniazid, Rifampicin, Pyrazinamide and Ethambutol for two months. All study subjects were followed up for 2 months after starting anti tubercular treatment and the response (decline in clinical symptoms and/or radiological clearing of pleural effusion) to treatment was seen at one month and two month. A final diagnosis was reached based on the composite reference standard and the study subjects were divided into two groups- TPE and non TPE with the following definitions: Tubercular pleural effusion was defined as an exudative pleural fluid (by Lights criteria) and positive pleural fluid ADA level and/or positive for AFB on microscopy along with response to anti - tubercular treatment. Pleural fluid analysis which did not meet the above criteria were considered as non - tubercular pleural effusion.

Statistical analysis:

We used SPSS software (version 16.0) to analyse the characteristics of the study population. Standard formulae were used to calculate the sensitivity, specificity, positive predictive values (PPV) and negative predictive values (NPV), positive likelihood ratios (LR+) and negative likelihood ratios (LR-). Mc-Nemar’s test was applied to compare variables. All comparisons were two-tailed. We considered the difference to be statistically significant if the P value was less than 0.05.

Results

We screened all consecutive patients with radiological evidence of pleural effusion during the study period and finally 158 of them were included in this study. (Figure 1)The baseline characteristics of the study subjects are summarized in Table 1.We observed that the mean age of the study subjects was 45 ± 17.3 years and that almost half of the study subjects were <40 years of age. There was male predominance with male to female ratio

being 3.4:1. Majority (78.5%) of study subjects had breathlessness as presenting symptom. Other presenting symptoms were cough of >15 days duration and fever in 62% each, pleuritic chest pain in 35.4%, loss of appetite in 27.2% and night sweats present in 3.2 %. None of the study subjects reported haemoptysis.

Fig 1. This is the Fig 1 Title "Flow chart of the study subjects".

Table 1. This is the Table 1 Title. "Baseline characteristics of study subjects".

Characteristic	Number (N=158)	Percentage
**Age (completed years)**		
12–40	73	46.2
41–60	51	32.3
60 and above	34	21.5
**Sex**		
Males	122	78.2
Females	36	21.8
**Symptoms of study subjects**		
Breathlessness	124	78.5
Cough	98	62
Fever	98	62
Pleuritic chest pain	56	35.4
Loss of appetite	43	27.2
Night sweats	5	3.2
**Nature of pleural fluid as per Light's criteria**		
Exudate	111	70.3
Transudate	47	29.7
**Adenosine Deaminase level in pleural fluid**		
Positive (>42 IU)	83	52.5
Negative (≥42 IU)	75	47.5
**Pleural fluid ZN microscopy for AFB**		
AFB Positive	0	0
AFB Negative	158	100
**Pleural fluid culture Mycobacteria Growth Indicator Tube liquid medium**		
MTB growth present	0	0
MTB growth absent	158	100
**Response to anti tubercular therapy (N=93)**		

Present ¶	90.¶	96.8¶
Absent¤	3 ¤	3.2¤
**Aetiology of pleural effusion determined by Composite Reference Standard¤**		
Tubercular.pleural.effusion ¶	90¶	56.9 ¶
Chronic.kidney.disease ¶	18¶	11.4¶
Congestive.cardiac.failure¶	14¶	8.9¶
Malignant.pleural.effusion¶	13¶	8.2¶
Cirrhosis.of.liver¶	12¶	7.6¶
Syn-pneumonic.effusion ¶	8¶	5.1¶
Pancreatic.effusion ¶	2¶	1.3¶
Chylothorax¤	1¤	0.6 ¤
**GeneXpert.MTB/RIF.assay.results.in.pleural.fluid¤**		
MTB.detected¶	15¶	9.5¶
MTB.not.detected¤	143¤	90.5¤
**Result.of.Rifampicin.resistance.test.in.pleural.fluid¤**		
Rifampicin.resistance.detected ¶	2¶	13.3¶
Rifampicin.resistance.not.detected¤	13¤	86.7¤

In our study we found that 70.3% (111/158) of the study subjects had exudative pleural effusion based on Light's criteria. Among these exudative pleural effusion 92.2 % (83/111) study subjects had positive ADA level, and 81% (90/111) were responded to anti tubercular therapy. Our study revealed pleural fluid ZN microscopy for AFB as well as growth on MGIT liquid culture medium was negative in all the study subjects. Based on the composite reference standard 57% of study subjects had TPE, 11.4% had chronic kidney disease, 8.2% had malignant pleural effusion, 7.6% had cirrhosis of liver, 9% had congestive cardiac failure, 5% had para-pneumonic effusion, 1.3% had pancreatic effusion and 0.65% had chylothorax as shown in table 1.

The GXpert assay gave a positive signal for the presence of MTB in 15/158 (9.5%) TPE study subjects. All these study subjects were also diagnosed to have TPE by composite reference standard. The sensitivity and specificity of the diagnostic test GXpert assay was found to be 16.6% (95%CI: 8.9 - 24.3) and 100% (95%CI: 97.2 - 100) respectively in diagnosing TPE. It

had a high PPV of 100% (95%CI: 88.3% - 100%) and a NPV of 52.4% (95%CI: 39.3% - 55.7%). The LR+ and LR-were 23.5 (95%CI: 1.4- 38.6) and 0.83 (95%CI: 0.76- 0.91) respectively. The diagnostic accuracy of GXpert assay was found to be 52.5% which was statistically significant as evidenced by p value < 0.05. (Table 2) Of the 15 study subjects in which MTB was detected in pleural fluid, rifampicin resistance was detected by GXpert assay in 2 (13.3%) study subjects.

Table 2. This is the Table 2 Title. Diagnostic measures of GXpert assay in dete[illegible]g tubercular pleural effusion as compared to Composite Reference Standard (N=158).

**Measure**	**Value**	**95% Confidence interval**
**Sensitivity**	16.6%	8.9-24.3
**Specificity**	100%	97.2-100
**Positive Predictive Value**	100%	88.3-100
**Negative Predictive Value**	52.4%	49.3-55.7
**Positive Likelihood ratio**	23.5	1.43-38.60
**Negative likelihood ratio**	0.83	0.76-0.91
**Diagnostic accuracy**	52.5%	
**P value**	< 0.05	

Discussion

In our study we found the sensitivity of the GXpert assay in diagnosing TPE to be 16.6% and the specificity to be 100%. The low sensitivity and high specificity was supported by a P value of < 0.05. These values were similar to earlier reports which found a low sensitivity and high specificity of

GXpert assay in detecting TPE as shown in table 3 (13-21). The previous studies reported low sensitivity with a wide range from 15% - 54.5% and all reported a very high specificity up to 100%. The low sensitivity of GXpert assay on pleural fluid could be explained by the low mycobacterial load leading to low DNA in pleural fluid. As expected the higher the mycobacterial load, it is more likely to obtain a positive result. Most of the studies conducted earlier reported a sensitivity similar to ours except for three studies. Rufai et al found the sensitivity of GXpert assay to be 54.5% which is higher than our study (14). Their study was conducted in a high TB burden area and included pleural fluid samples of patients who were TB suspects. Another study conducted by Du et al found the sensitivity of GXpert assay to be 43.6% which is also relatively higher than in our study (16). In addition to pleural fluid sample, they used pleural biopsy specimens as well which may have resulted in a relatively higher sensitivity. Trajman et al in their study found the sensitivity of GXpert assay to be 3%, which very low as compared to our study (17). The possible reason for this could be the presence of inhibitory substances in the pleural fluid as they did not submit the clinical specimens to any processing before running the test. Blood and other inhibitors, such as heparin or pus which are present in the collected sample could have interfered with cell lysis and cause inactivation of the DNA polymerase resulting in false-negative results. Also they did not use fresh pleural fluid sample and the long storage time in freezers could explain the low sensitivity.

Table 3. This is the Table 3 Title. Comparison with previous studies conducted on the diagnostic utility of Tuberculosis assay in detecting Tubercular pleural effusion.

**Sr. No**	**Author (Reference)/Year**	**Country**	**Study Type/ Sample Size**	**Mean age (± SD) years**	**Sex Ratio (M:F)**	**Sensitivity (%), Specificity (%). (95% CI)**	**PPV (%), NPV (%). (95% CI)**	**LR+, LR -. (95% CI)**	**P. value**
**1**	Our Study/2018	India	Cross sectional/158	45±17.33	3.4:1	16.6(8.9-24.3), 100(97.2-100)	100(88.3-100), 52.4 (49.3-55.7)	23.5(1.4-38.6) 0.83(0.76-0.91)	<0.05
**2**	Rufai(14)/2015	India	Prospective Observational/162	39±18	3:1	54.8 (38-70), 100 (85-100)	-	-	<0.01
**3**	Sehgal(13)/2014	India	Meta-analysis/24	-	-	22.7, 51.4	-	-	-
**4**	Denkinger(21)/2013	USA	Meta-analysis/841	-	-	21.4 (8-33), 98.7 (89-100)	-	-	-
**5**	Lusiba(18)/2013	Uganda	Cross sectional/116	34±13	1:1	28.7, 96.6	96.1,31.1	-	<0.05
**6**	Trajman(17)/2013	Brazil	Cross sectional/203	45±7.5	3:1	3 (0-17), 100 (89-100)	-	-	0.02
**7**	Porcel(20)/2012	Spain	Case Control/67	33	2:1	15 (7-32), 100 (88-100)	-	11.3, 0.85	0.30
**8**	Du (16)/2012	China	Cross sectional/134	38.6±12.3	1.25:1	43.6,98.6%	96,69.3	-	<0.01
**9**	Friedrich(15)/2011	South Africa	Cross sectional/25	-	-	25,100	-	-	<0.001
**10**	Meldau (19)/2012	South Africa	Prospective Cohort/103	39±10	3:2	22.5 (12.4-37), 98 (89-99.7)	91.4, 69.7	-	<0.05

Our study demonstrates that a negative GXpert assay does not completely exclude the diagnosis of TPE as the test was unable to identify 83.4% (100-16.6) of confirmed TPE cases by composite reference standard (low sensitivity) and when GXpert assay was negative, 52.4% of patients still had TPE (low negative predictive value). These findings suggest that clinical acumen in combination with routine pleural fluid analysis should be the key factor in the diagnosis of TPE in clinically and radiologically suspected patients, especially in high TB burden countries.

Pleural fluid GXpert assay to diagnose TPE has been evaluated in very few studies in India. The major strength of our study is use of standardized protocol and an adequate sample size. We have followed up the patients to see the clinical response of anti-tubercular treatment and thus confirmed our diagnosis which has not been done in any of the previous studies. Also the sample collection was done under stringent measures and immediate transportation to the laboratory without any time lag was done, improving the chances of a positive test as a time delay in initiating the test or use of frozen pleural fluid sample could lead to false negative results.

The study had a few limitations. To reach a final diagnosis of TPE we used a composite reference standard as opposed to the gold standard which includes histopathological demonstration of AFB in pleural tissue or a growth of MTB on culture medium. To conduct a pleural biopsy in all patients was not feasible in our setting and although we did use pleural fluid culture, its results were limited due to a poor diagnostic yield. We did not perform some of the tests that are known to be indicative of TPE including markers such as Interferon gamma assays because these are not readily available in our setting.

A multicentric study with larger sample size including patients varying TB burden could help achieve a more accurate result. Also further evaluation of diagnostic algorithms in different epidemiological and geographical settings and patient populations can be undertaken. Its cost-effectiveness and cost-benefit analyses along with its impact on the diagnosis of TB, multidrug resistant TB and the management of patients can be studied.

Conclusion

In the light of our findings we have reached to the conclusion that GXpert assay has a very high specificity in diagnosing TPE but has a low sensitivity. Thus its clinical utility is limited when used as a standalone test. A physician's clinical acumen in combination with routine pleural fluid analysis should be the key factor in the diagnosis of TPE in clinically and radiologically suspected patients, especially in high TB burden countries.

Acknowledgments

We wish to thank our patients for their cooperation in this study.

Author Contributions

Conceived and designed the experiments: JJ PJ. Performed the experiments: JJ PJ. Analyzed the data: JJ SB PS. Contributed reagents/materials/analysis tools: JJ PJ SB PS. Wrote the paper: JJ PJ SB PS.

References

1. Programme WHOGT. Automated real-time nucleic acid amplification technology for rapid and simultaneous detection of tuberculosis and rifampicin resistance : Xpert MTB/RIF assay for the diagnosis of pulmonary and extrapulmonary TB in adults and children 2013. Available from: http://www.ncbi.nlm.nih.gov/books/NBK258608/.
2. Skouras VS, Magkouta SF, Psallidas I, Tsilioni I, Maragozidis P, Gourgoulianis KI, et al. Interleukin-27 improves the ability of adenosine deaminase to rule out tuberculous pleural effusion regardless of pleural tuberculosis prevalence. Infectious diseases (London, England). 2015;47(7):477-83.
3. SV S, AR E, G F. - Transmission of drug-susceptible and drug-resistant tuberculosis and the critical. Clin Infect Dis. 2010;15(50):651496.
4. SK S, A M. - Extrapulmonary tuberculosis. Indian J Med Res. 2004;120(4):316-53.
5. D H, M J, E S, C B, E W, K H, et al. - Rapid detection of Mycobacterium tuberculosis and rifampin resistance by use of. J Clin Microbiol. 2010;48(1):229-37.
6. D H, S R-G, C B, E R. - Rapid molecular detection of extrapulmonary tuberculosis by the automated. J Clin Microbiol. 2011;49(4):1202-5.
7. A T, C K, RR L, RD S, ML R, MH FS, et al. - Pleural fluid ADA, IgA-ELISA and PCR sensitivities for the diagnosis of pleural. Scand J Clin Lab Invest. 2007;67(8):877-84.
8. V V, C B, P N, A S, D A, C R. - Xpert MTB/RIF: a new pillar in diagnosis of extrapulmonary tuberculosis? J Clin Microbiol. 2011;49(7):2540-5.
9. Programme WHOGT. Xpert MTB/RIF implementation manual : technical and operational 'how-to': practical considerations 2014. Available from: http://www.ncbi.nlm.nih.gov/books/NBK254323/.
10. S A, P V, G D, R C, N L. - Comparison of the Xpert MTB/RIF test with an IS6110-TaqMan real-time PCR assay. J Clin Microbiol. 2011;49(5):1772-6.
11. SD L, MP N. - Xpert(R) MTB/RIF assay: development, evaluation and implementation of a new rapid. Future Microbiol. 2011;6(9):1067-82.
12. Y L, Y P, T Z, X X, X W, J Y, et al. - Rapid diagnosis of extrapulmonary tuberculosis with Xpert Mycobacterium. J Med Microbiol. 2017;66(7):910-4.
13. IS S, S D, AN A, D B, R A. - Diagnostic Performance of Xpert MTB/RIF in Tuberculous Pleural Effusion. J Clin Microbiol. 2016;54(4):1133-6.
14. SB R, A S, P K, J S, S S, http://orcid.org I-O. - Performance of Xpert MTB/RIF Assay in Diagnosis of Pleural Tuberculosis by Use of. J Clin Microbiol. 2015;53(11):3636-8.

15. SO F, F vG-B, AH D. - Xpert MTB/RIF assay for diagnosis of pleural tuberculosis. J Clin Microbiol. 2011;49(12):4341-2.
16. J D, Z H, Q L, G X, X X, W L, et al. - Rapid diagnosis of pleural tuberculosis by Xpert MTB/RIF assay using pleural. J Res Med Sci. 2015;20(1):26-31.
17. A T, EF dSSKdO, ML B, E BN, EM S, MC dSL, et al. - Accuracy of polimerase chain reaction for the diagnosis of pleural tuberculosis. Respir Med. 2014;108(6):918-23.
18. JK L, L N, BJ K, A K, R L, M N, et al. - Evaluation of Cepheid's Xpert MTB/Rif test on pleural fluid in the diagnosis of. PLoS One. 2014;9(7):0102702.
19. R M, J P, G T, G C, B A, G S, et al. - Comparison of same day diagnostic tools including Gene Xpert and unstimulated. BMC Pulm Med. 2014;14(58):1471-2466.
20. JM P, R P, L V, S B, E S-J, A E. - Xpert(R) MTB/RIF in pleural fluid for the diagnosis of tuberculosis. Int J Tuberc Lung Dis. 2013;17(9):1217-9.
21. CM D, SG S, CC B, N D, M P, KR S. - Xpert MTB/RIF assay for the diagnosis of extrapulmonary tuberculosis: a. Eur Respir J. 2014;44(2):435-46.

Supporting information

SI Fig. This is the S1 Fig Title. This is the S1 Fig legend.

Click here to access/download;Figure;Fig 1.tif

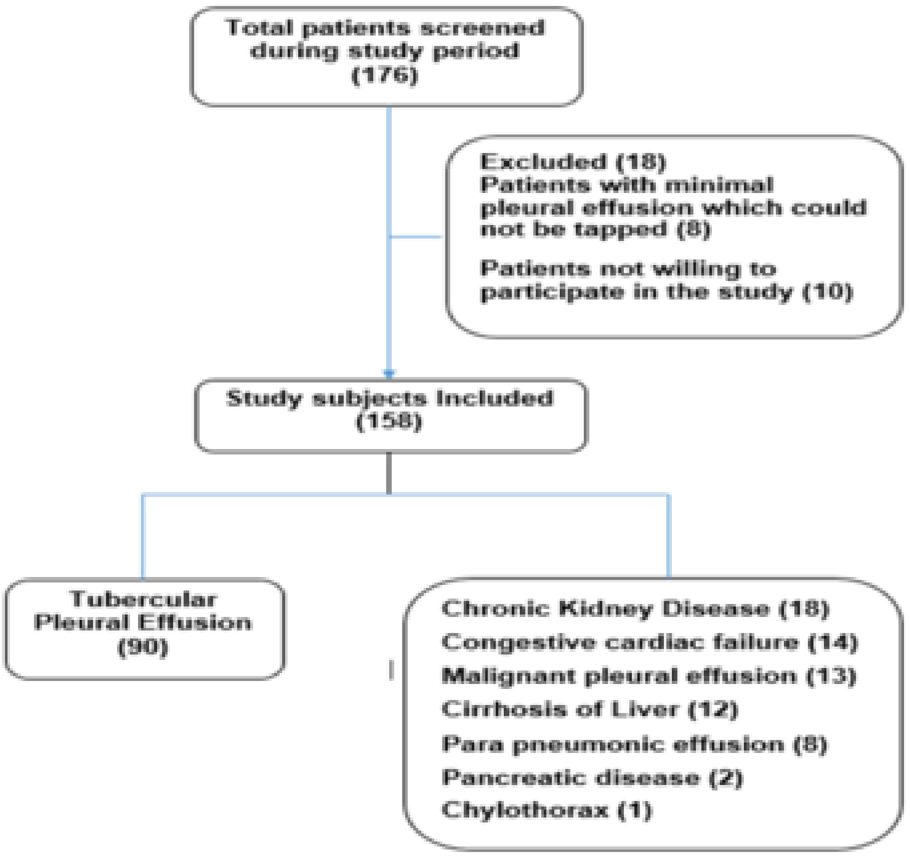

Supplement: Attachment — Submitted filename: PONE-D-19-33113_hds.pdf [file pone.0251618.s002.pdf]
