# Supplementary material for: Diagnostic accuracy of GeneXpert MTB/RIF assay for detection of tubercular pleural effusion
Source: PLoS One. 2021 Jun 14;16(6):e0251618. doi: 10.1371/journal.pone.0251618 (PMC8202948; doi:10.1371/journal.pone.0251618)
Supplement: Attachment — Submitted filename: response to reviewer.docx [file pone.0251618.s003.docx]

The authors would like to thank the academic editor and reviewers for their specific and helpful comments.

**Manuscript no:** PONE-D-19-33113

The manuscript has been improved according to the suggestions:

Editor comments-

1) We have ensured that manuscript meets PLOS ONE’s style requirements and have made the necessary corrections to our manuscript.
2) Regarding the occurrence of overlapping text with previous publication we have rephrased the sentence and cited the original source.
3) As per your suggestion we will change to our Data Availability statement, in our cover letter.
4) Supporting information S1 figure has been uploaded after edited through Preflight Analysis and Conversion Engine (PACE) digital diagnostic tool.
5) We restructured the introduction as per your suggestion . and mentioned the national TB programme of country .
6) We have explained the recruitment process and sample size calculation in detail.
7) In the Discussion section we have provided key policy and practice recommendations for national programme based on the results of our study.
8) Line 61- We have clarified the need for this study by adding the following points- ‘although few studies have been conducted in this area, there has been a wide variation in the findings in all the studies’

**Minor comments:**

1. We introduced all abbreviations when mentioned first along with the full form in parenthesis.
2. Line 78-79 is a repeat of Line 73-75, so it was deleted.
3. Line 79-81 changed as part of study participants.
4. Line 117- 118 we have made the correction and the value of pleural fluid ADA > 42IU/L was finalized according to our central laboratory.
5. Page 14, Line 25-26 are deleted
6. Page 13, Line 7: Removed as a major strength of the study as it is part of methodology.

We have mentioned that the results of our study is not generalizable to other contexts under Limitations, considering this study was performed in one center of the country.
**Reviewer #1:**

**Major comments:**

1) We have restructured our introduction as per your suggestion.
2) The recruitment process is explained in the revised manuscript along with the detailed method for sample size calculation.
3) We have provided key policy and practice recommendations in discussion part as suggested.
1) **Minor comments:**
    Reference style and reference lists has been updated.
2) We have mentioned abbreviations in parenthesis at the first mention, have made the
3) necessary corrections in tables and modified the strenghts of the study.
4) Table 1: As suggested by the reviewer, we changed the overlap in age categories: from 60
5) and above to 61 and above.
6) The title has been changed as per your suggestions for Table 1. We have also added footnote.
7) We made the changes in line numbers, now they are continuous.

**Reviewer #2:**
Table 1 –Addition of 2x2 contingency table for better clarity as suggested has been added.

| **GeneXpert MTB/RIF** | **Composite reference standard** | | **Total** | **P value** |
| --- | --- | --- | --- | --- |
|  | **Positive** | **Negative** |  |  |
| **Positive** | 15 | 0 | 15 | **<0.05** |
| **Negative** | 75 | 68 | 143 |  |
| **Total** | 90 | 68 | 158 |  |

1) Line 182-189 and table 3: we have incorporated gold standard of other studies advised.
2) Line 8-9, page 13- Of the total 158 study subjects 93 were started on anti-tubercular therapy based on clinical findings, exudative pleural effusion by Light’s criteria and positive pleural fluid ADA level. Of the 93 subjects, 90 (96.7%) responded to anti tubercular treatment while remaining 3 subjects did not respond.
3) Line 24, page 14- the correction suggested has been incorporated in our manuscript.
4) Reference style has been changed according to PLOS ONE guidelines.

Reviewer 2 comments-

1) Detailed method for sample size calculation along with sensitivity assumed in each group is mentioned in revised manuscript.we have compared Xpert MTB/RIF to composite reference to calculate various parameters of diagnostic study.
2) We have corrected the error in negative predictive value and positive likelihood ratio as suggested by you.
3) In conclusions section we have mentioned about rifampicin resistant as suggested by you.
4) We have mentioned that first two authors have contributed equally and the third and fourth authors have contributed equally to the work done.
5) We introduced all abbreviations, reference and corrected grammatical mistakes

We hope that our modifications render our manuscript in its current form suitable for publication in PLOS ONE.

Yours sincerely,

On behalf of the authors,

Dr Jyoti Jain
