# Supplementary material for: Diagnostic accuracy of GeneXpert MTB/RIF assay for detection of tubercular pleural effusion
Source: PLoS One. 2021 Jun 14;16(6):e0251618. doi: 10.1371/journal.pone.0251618 (PMC8202948; doi:10.1371/journal.pone.0251618)
Supplement: Attachment — Submitted filename: Response to Reviewers.docx [file pone.0251618.s004.docx]

14^th^ of August 2020

The authors would like to thank the academic editor and reviewers for their specific and helpful comments.

Please find enclosed the edited manuscript in word format with track changes.

**Title**: Diagnostic accuracy of GeneXpert MTB/RIF assay for detection of tubercular pleural

Effusion.

**Author:** Jyoti* Jain, Pooja$ Jadhao, Shashank^ Sharadchandra Banait, Preetam# Salunkhe.

**Manuscript no:** PONE-D-19-33113

The manuscript has been improved according to the suggestions:

**ACADEMIC EDITORS ADDITIONAL COMMENTS**

Editor Comments 1: line 61 - needs a review. Check for studies on Xpert and EP samples.

Please look at your table 3. There are studies. Please provide a summary of the table here and mention what is 'new' in your study? 'Why' this study? Purpose of the study

AUTHOR RESPONSE: Line 61- We have clarified the need for this study by adding the following points- ‘although few studies have been conducted in this area, there has been a wide variation in the findings in all the studies’. Page 4, Line 64-68.

Editor Comments 2: Line 73-75, not part of settings.

AUTHOR RESPONSE: Line 73-75 are removed from setting.

Editor Comments 3: Line 92- check the sentence

AUTHOR RESPONSE: Line 92 sentence restructured. Page 6, Line 106.

Editor Comments 4: Line 117 ref

AUTHOR RESPONSE: Line 117- 118 we have made the correction and the value of pleural fluid ADA > 42IU/L was finalized according to our central laboratory. Page 7, Line 131-132.

Editor Comments 5: line 119-120 ref

AUTHOR RESPONSE: Reference added and sentence restructured. Krishna M, Gole SG. Comparison of conventional Ziehl–Neelsen method of acid fast bacilli with modified bleach method in tuberculous lymphadenitis. Journal of cytology. 2017 Oct;34(4):188.

Editor Comments 6: Tables and figure titles, Please ensure that the titles are standalone. Ensure a time, place person component in title. Add appropriate footnotes.

AUTHOR RESPONSE: The title has been changed as per your suggestions for Tables and figures. Appropriate footnotes added.

Editor Comments 7: Table 1. Use meaningful age cut offs

say <10

10-19

20 and above

OR

<15

15-44

45-64

65 and above

AUTHOR RESPONSE: In our hospital setting age group upto 12 years are admitted in paediatrics department and in the department of medicine we cater patients with ≥ 12 years of age. As this study included patients from department of medicine we have kept these age cut offs.

Editor Comments 8: In addition to the table 2 presented by the authors,

Show the standard two by two table as a separate table.

Column heading - TPE+ TPE-

Row heading - Xpert+ XPert-

AUTHOR RESPONSE: 2x2 contingency table for better clarity as suggested has been added. Page 11, Line 179-180.

| **GeneXpert MTB/RIF** | **Composite reference standard** | | **Total** | **P value** |
| --- | --- | --- | --- | --- |
|  | **TPE Positive** | **TPE Negative** |  |  |
| **Positive** | 15 | 0 | 15 | **<0.05** |
| **Negative** | 75 | 68 | 143 |  |
| **Total** | 90 | 68 | 158 |  |

Editor Comments 9: Authors used a composite index for TPE and used it as a gold standard.

What was the gold standard used in these studies?

AUTHOR RESPONSE: We have incorporated gold standard of other studies as advised in Table 3.

Editor Comments 10: Table 3. Please also add a column heading and mention what gold standard was used in the respective studies?

AUTHOR RESPONSE: In table 3, we have incorporated gold standard of other studies as advised.

Editor Comments 11: Line 8-9, page 13.almost all with TPE+ (based on composite index) responded to ATT. This line has to be specifically there in results narrative (give numerator and denominator)

AUTHOR RESPONSE: Of the total 158 study subjects 93 were started on anti-tubercular therapy based on clinical findings, exudative pleural effusion by Light’s criteria and positive pleural fluid ADA level. Of the 93 subjects, 90 (96.7%) responded to anti tubercular treatment while remaining 3 subjects did not respond. Page 4, Line 64-68.

Editor Comments 12: Line 10, page 13

low time lag = is this supported by data?

AUTHOR RESPONSE: Yes, this is supported by study done by [Friedrich](https://www.ncbi.nlm.nih.gov/pubmed/?term=Friedrich%20SO%5BAuthor%5D&cauthor=true&cauthor_uid=21998430) et al published in J Clin Microbiol in 2011. According to this study statistically significant difference (P= 0.0021, paired two tailed Student’s *t* test)is found due to storage of sample and frozen samples showed false negative results for M tuberculosis by Xpert assay. We are adding reference to this sentence also. Page 16, Line 222.

Editor Comments 13: Line 24, page 14

Limited clinical utility as a standalone test expecially if the Xpert results are negative (specify this)

AUTHOR RESPONSE: Line 24, page 14- the correction suggested has been incorporated in our manuscript.

**REVIEWER #1: MAJOR COMMENTS**

REVIEWER COMMENT1: Introduction needs restructuring. The reference to India has been provided in beginning of the introduction, however, no further mention of national TB programme of country or relevance to country is given. The authors can strengthen the rationale of the study, if it is aimed towards India or for global evidence.
AUTHOR RESPONSE: We have restructured our introduction as per your suggestion and mentioned the national TB programme of country. Page 3, Line 49-53, Page 4, Line 64-68.

REVIEWER COMMENT 2: The recruitment process must be explained in the manuscript. If sample size was calculated as 158, how did the researchers stop the recruitment once it reached the sample size.
AUTHOR RESPONSE: The recruitment process is explained in the revised manuscript along with the detailed method for sample size calculation. Page 5, Line 88-100.

REVIEWER COMMENT3: Discussion: The authors must provide key policy and practice recommendations for national programme or at global level, based on the results.
AUTHOR RESPONSE: We have provided key policy and practice recommendations in discussion part as suggested. Page 16, Line 230-232.

**REVIEWER #1: MINOR COMMENTS**
REVIEWER COMMENT 1: Reference style and reference lists must be updated. For example, Reference 2, 3 does not reflect the statement in manuscript. References do not have complete title of the manuscripts. Please review
AUTHOR RESPONSE: Reference style and reference lists has been updated. Page 17-18, Line 249-293.

REVIEWER COMMENT 2: Abbreviations must be mentioned first along with the full form in parenthesis. Line 46 mentioned TPE, however it must be mentioned in Line 42 first.
AUTHOR RESPONSE: We introduced all abbreviations when mentioned first along with the full form in parenthesis. Page 3, Line 39.

REVIEWER COMMENT 3: Line 78-79 is a repeat of Line 73-75. Please review
AUTHOR RESPONSE: Line 78-79 is a repeat of Line 73-75, so it was deleted.

REVIEWER COMMENT 4: Line 79-81 could be mentioned under Study participants, and not Study design.

AUTHOR RESPONSE: Line 79-81 deleted from study design.

REVIEWER COMMENT 5: Line 117-118 needs a reference.

AUTHOR RESPONSE: Line 117- 118 we have made the correction and the value of pleural fluid ADA > 42IU/L was finalized according to our central laboratory. Page 7, Line 132-133.

REVIEWER COMMENT 6: The title of figures and tables must be stand-alone. It must include information about the study site and time period.
AUTHOR RESPONSE: The title has been changed as per your suggestions for Tables and figures.

REVIEWER COMMENT 7: Table 1: o There is an overlap in age categories: It must be 61 and above and not 60 and above. o Symptoms of study subjects: It is understood that each patient may report one or more than one symptom. This must be mentioned in the footnote of the table.
AUTHOR RESPONSE: Table 1: As suggested by the reviewer, we changed the overlap in age categories: from 60 and above to 61 and above. We have also added footnote to Table 1 as per your suggestion. Page 9, Table 1.

REVIEWER COMMENT 8: Line numbers are not continuous, the line numbers have restarted from Page 13. Please review
AUTHOR RESPONSE: We made the changes in line numbers, now they are continuous.

REVIEWER COMMENT 9: Page 14, Line 25-26 are repeat of Page 13 Line 4-5. Please edit.
AUTHOR RESPONSE: Page 14, Line 25-26 is a repeat of Page 13 Line 4-5, so it was deleted.

REVIEWER COMMENT 10: Page 13, Line 7: Not sure if the use of standardized protocol and adequate sample size is a major strength of the study. It is part of methodology and a study must follow the protocol.
AUTHOR RESPONSE: Page 13, Line 7: Removed as a major strength of the study as it is part of methodology.

REVIEWER COMMENT 11: The authors may add that the study is not generalizable to other contexts under Limitations, considering this study was performed in one center of the country.

AUTHOR RESPONSE: We have mentioned that the results of our study is not generalizable to other contexts under Limitations, considering this study was performed in one center of the country. Page 16, Line 228-229.

**REVIEWER #2: MAJOR COMMENTS**

REVIEWER COMMENT 1: The article is important because it provides evidence on the sensitivity of Xpert MTB/RIF assay among Extrapulmonary TB (EPTB). The End TB strategy targets all types of TB and not only bacteriologically confirmed, therefore knowing the sensitivity and predictive values of Xpert MTB/RIF is key to reach the true patients suffering from (EPTB) and allowing reaching targets of End TB strategy. Also, the study is performed in a high TB burden country with limited resources. Those characteristics are common in high burden countries. In that sense, the study suggests a composite score for diagnosing EPTB that could be very useful in similar contexts.
AUTHOR RESPONSE: Detailed method for sample size calculation along with sensitivity assumed in each group is mentioned in revised manuscript. Page 5, Line 88-100.

REVIEWER COMMENT 2: There is room to improve the quality of the article. I suggest to better explain the calculation of sample size. Were the two groups, one Xpert MTB/RIF were compared to composite? if yes, what was the sensitivity assumed in each group?
AUTHOR RESPONSE: Detailed method for sample size calculation is mentioned in revised manuscript as per reviewer suggestion. We have compared Xpert MTB/RIF to composite reference to calculate various parameters of diagnostic study. Page 5, Line 88-100.

REVIEWER COMMENT 3: In the results part, the negative predictive value (NPV) the figure should be revised. If there were 68 true negatives divided by true negatives plus false negatives (75), NVP should be 47.55% and not 52.4%. If the numbers are provided aside to the percentage this could improve the understanding of the calculation. Then, the likelihood (Sensitivity/1-Specificity) positive ratio would be -0.83 and not 23.5. These issues need to be revised.
AUTHOR RESPONSE: We have corrected the error in negative predictive value and positive likelihood ratio as suggested by you. Page 13, Table 3 Row 5 and 6.

REVIEWER COMMENT 4: Concerning the clinical response, it would be useful to have the treatment outcomes at the end of full treatment completion.
AUTHOR RESPONSE: I also agree with your opinion regarding clinical response to the treatment at the end of full treatment completion. However being a cross sectional observational study, we did not followed up the study participants for 6months however among patients in whom Anti tubercular treatment was started they were followed up to 2 months of intensive phase of treatment. Page 16, Line 219.

REVIEWER COMMENT 5: In conclusions: nothing is mentioned about rifampicin resistant diagnosed patients, which was one of the objectives of the study.
AUTHOR RESPONSE: In conclusions section we have mentioned about rifampicin resistant as suggested by you. Page 17, Line 240.

**REVIEWER #2: FORMAT COMMENTS**

REVIEWER COMMENT 1: Line 3: Capital in ‘jain’ = Jain

AUTHOR RESPONSE: Changes done as per suggestion. Page 1, Line 3.

REVIEWER COMMENT 2: Line 11: no need to repeat ‘& These authors…” because it seems that all authors contributed equally.

AUTHOR RESPONSE: ¶These authors contributed equally to this work (means Jyoti Jain and Dr Pooja Jadho)

& These authors also contributed equally to this work. (means Shashank Banait and Preetam salunkhe)

REVIEWER COMMENT 3: Line 58: superscript ‘11’ not clear if it is reference as the references are formatted in brackets.
AUTHOR RESPONSE: Changes done as per suggestion and reference is formatted as per PLoS guidelines.

REVIEWER COMMENT 4: In tables: there are full stops in name of categories that should be removed.
AUTHOR RESPONSE: In tables full stops in name of categories are removed as suggested by reviewer.

REVIEWER COMMENT 5: Reference 12 is cited as WHO when the source comes from J Med Microbiol.

AUTHOR RESPONSE: Changes done as suggested by reviewer.

We hope that our modifications render our manuscript in its current form suitable for publication in PLOS ONE.

Yours sincerely,

On behalf of the authors,

Dr Jyoti Jain
