# Supplementary material for: Diagnostic accuracy of GeneXpert MTB/RIF assay for detection of tubercular pleural effusion
Source: PLoS One. 2021 Jun 14;16(6):e0251618. doi: 10.1371/journal.pone.0251618 (PMC8202948; doi:10.1371/journal.pone.0251618)
Supplement: Attachment — Submitted filename: response to reviewer 24 2 21.docx [file pone.0251618.s005.docx]

24^th^ of February 2021

The authors would like to thank the academic editor and reviewers for their specific and helpful comments.

Please find enclosed the edited manuscript in word format with track changes.

**Title**: Diagnostic accuracy of GeneXpert MTB/RIF assay for detection of tubercular pleural

Effusion.

**Author:** Jyoti* Jain, Pooja$ Jadhao, Shashank^ Sharadchandra Banait, Preetam# Salunkhe.

**Manuscript no:** PONE-D-19-33113R3

The manuscript has been improved according to the suggestions:

**MINOR COMMENTS**

COMMENT 1:  Please upload a copy of Supporting Information S1 Figure which you refer to in your text on page 17.
AUTHOR RESPONSE: We are uploading a copy of Supporting Information S1 Figure along with this revision.

COMMENT 2: We note that you currently have two Tables in your manuscript titled as Table 3. The first "Table 3 : Diagnostic measures of GXpert assay in detecting tubercular pleural effusion as compared to Composite Reference Standard (N=158) - and the second "Table 3: Comparison with previous studies conducted on the diagnostic utility of Tuberculosis assay in detecting Tubercular pleural effusion."
So that these tables can be differentiated can you please update the Table title numbering and the in-text citations to them accordingly.

AUTHOR RESPONSE: We have changed the Table title numbering and the in-text citations to them accordingly as per your suggestion. Page 12 line 199, Page 14, Line 216.

We hope that our modifications render our manuscript in its current form suitable for publication in PLOS ONE.

Yours sincerely,

On behalf of the authors,
